# LAMP2: a major update of the database linking antimicrobial peptides

**DOI:** 10.1093/database/baaa061

**Published:** 2020-08-25

**Authors:** Guizi Ye, Hongyu Wu, Jinjiang Huang, Wei Wang, Kuikui Ge, Guodong Li, Jiang Zhong, Qingshan Huang

**Affiliations:** 1 State Key Laboratory of Genetic Engineering, School of Life Sciences, Fudan University, Shanghai 200438, China; 2 Kunshan Bio-Green Biotechnology Co., Ltd, Kunshan 215316, Jiangsu, China; 3 Shanghai High-Tech United Bio-Technological R&D Co., Ltd, Shanghai 201206, China

**Keywords:** antimicrobial peptides, database, LAMP

## Abstract

Antimicrobial peptides (AMPs) have been regarded as a potential weapon to fight against drug-resistant bacteria, which is threating the globe. Thus, more and more AMPs had been designed or identified. There is a need to integrate them into a platform for researchers to facilitate investigation and analyze existing AMPs. The AMP database has become an important tool for the discovery and transformation of AMPs as agents. A database linking antimicrobial peptides (LAMPs), launched in 2013, serves as a comprehensive tool to supply exhaustive information of AMP on a single platform. LAMP2, an updated version of LAMP, holds 23 253 unique AMP sequences and expands to link 16 public AMP databases. In the current version, there are more than 50% (12 236) sequences only linking a single database and more than 45% of AMPs linking two or more database links. Additionally, updated categories based on primary structure, collection, composition, source and function have been integrated into LAMP2. Peptides in LAMP2 have been integrated in 8 major functional classes and 38 functional activities. More than 89% (20 909) of the peptides are experimentally validated peptides. A total of 1924 references were extracted and regarded as the evidence for supporting AMP activity and cytotoxicity. The updated version will be helpful to the scientific community.

## Introduction

During the past 30 years, antimicrobial peptides (AMPs) have been considered as a potential source for the development of new antibacterial drugs against drug-resistant bacteria (1, 2). Currently, a lot of potential AMPs have been designed or identified. But most of them have failed in clinical trials. A thorough understanding of the role of sequence of AMPs on their specificity and activity is essential to exploit them as antimicrobial agents. The AMP database has become an important tool for the discovery and transformation of AMPs (3). Now, there are more and more AMP databases that had been developed. Some of them are general databases, such as APD (4), DBAASP (5), CAMP (6) and dbAMP (7), whereas others are specialized databases, such as CancerPPD, Hemolytik, THPdb, InverPep and AntiTbPdb. CancerPPD (8) is a database of anticancer peptides and proteins, Hemolytik (9) is a database of experimentally determined hemolytic and non-hemolytic peptides, THPdb (10) is a database of FDA-approved peptide and protein therapeutics, InverPep (11) is a database of invertebrate AMPs and AntiTbPdb is a database of experimentally verified anti-tubercular or anti-mycobacterial peptides.

**Figure 1:**
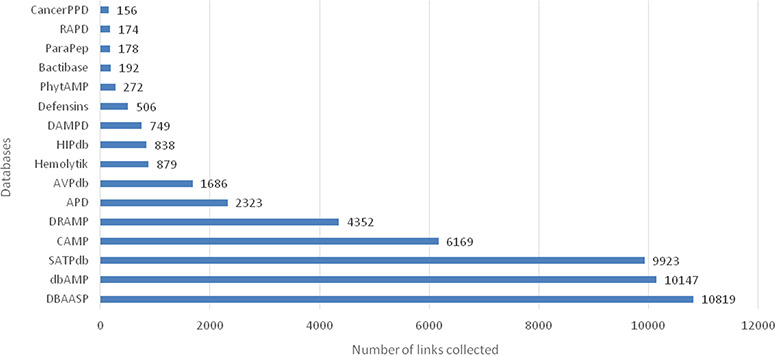
A total of 23 253 entries are distributed in 16 different databases in LAMP2.

**Table 1:** Comparison of data in two versions of LAMP database

Group		LAMP	LAMP2
By primary structure	Linear	/	22 934
	Cyclic		319
By collection	Experimental validated peptides	3203	20 909
	Patent peptides	1491	1491
	Predicted peptides	853	853
By composition	Peptides having D-amino acids	/	839
	Peptides having natural amino acids		21 691
	Peptides having modified residues		723
By source	Synthetic peptides	1643	15 429
	Natural peptides	3904	7824

The increase in the number of databases is on the one hand, the increase in the amount of AMP sequences is on the other. For example, the number of records in CAMP was about 3782 in the 2009 version (12). In 2013, the number of records exceeds 6756 (13). And in 2015, the number of records exceeds 10 000 (6). It is too difficult for researchers to obtain all the properties of AMPs from dozens of various databases and tens of thousands of sequences. As of date, the most entries databases, such as dbAMP (12 389 AMP sequences), CAMP_R3_ (10 247 AMP sequences) and SATPdb (10 585 AMP sequences) (14), have their own characteristics or limitations. It creates a need to establish a comprehensive data platform integrating the latest AMP database information. LAMP, a database linking AMP, serves as a tool to provide a full collection of AMPs with cross-linking between existing databases. Compared with other available AMP databases, significant improvements available in LAMP include not only significantly more AMPs but also the unique cross-linking and top similar AMP (Topview) functions. LAMP is a comprehensive platform integrating majority of the peptides from various databases. Since the original version of LAMP was released online in 2013 (15), LAMP has been widely accepted and utilized. In the past, a significant number of AMPs have been discovered. It is proper to include the newly identified AMPs into the updating LAMP. Here, we present LAMP2, an update to the existing LAMP database. LAMP2 would help researchers work on AMPs more efficiently and conveniently.

## Materials and Methods

### Data collection and compilation

All the AMP sequences were collected from the scientific literatures or authoritative public AMP-related databases. In this updated LAMP2, the public AMP databases contain 16 public databases (4–9, 14, 16–24). We obtained AMP information using export option provided by databases or using the ‘wget’ program. Then, we selected identical AMP sequences of less than 100 amino acid residues long exclude the existing AMPs in previous LAMP database and integrated them into the LAMP2 platform after annotating the physicochemical properties, activities, functions, references and other basic information for each AMP. Structural information of AMPs was retrieved from protein databases of PDB. A total of 1924 references were extracted and regarded as the evidence for supporting AMP activity and cytotoxicity. All sequences of AMPs are presented in FASTA format.

## Results and Discussion

### Data update

#### Sequences and links to databases update

After manual checking and removal of redundancy, the current updating LAMP2 holds 23 253 unique AMP sequences. The cross-linking databases expand to 16 different published AMP databases including the latest or the most commonly used AMP databases, such as APD, CAMP, DBAASP, dbAMP (2019), SATPdb, DRAMP (2019) (Figure 1). Compared with the previous version, LAMP2 has added almost 20 000 new entries (Table 1). Most entries in LAMP2 are cross-linked with other individual databases. The cross-linking databases comprised 16 public AMP databases, which are nearly three times of the previous version.

**Table 2:** New categories based on functional activity in LAMP2

Function classes	Functional activity	Numbers of AMPs
Antibacterial	Antibacterial	15 844
	Anti-Gram positive	7026
	Anti-Gram negative	7101
Antifungal	Antifungal	2147
Antiviral	Antiviral	4241
Antiparasitic	Antiparasitic	374
New functional peptides	Wound healing	19
	Antioxidant	22
	Spermicidal	13
	Enzyme inhibitor	25
	Mollicute	16
	Antihypertensive	15
Disease-associated peptides	Anti-MRSA	114
	Anti-HIV	105
	Anti-inflammatory	2
	Anticancer	1314
	Anti-tumor	22
New mechanism-associated peptides	Chemotactic	50
	Sodium channel blocker	2
	Surface immobilized	10
	Anti-biofilm	34
	Cancer cells	223
	Mammalian cells	3682
	Blood-brain barrier	10
	Cell penetrating	12
	Tumor homing	2
	Hormones	3
	Quorum sensing	5
Toxic	Hemolytic	1601
	Cytotoxic	244

#### Categories of entries update

To meet the needs of different researchers for different options, the categories of updated database are more abundant. We added two types of new classification and expand the activity group. The new categories were based on primary structures and the composition of special amino acid. LAMP2 include linear (22934) and cyclic (319) based on primary structures, peptides having D-amino acids (839), peptides having natural amino acids (21691) and peptides having modified residues (723) based on the composition (Table 1). The section of functional update list expanded groups.

#### Functional update

Based on curated information of functional activity in 16 databases, peptides in LAMP2 have been integrated in 8 major functional classes and 38 functional activities (Table 2). The maximum numbers of peptides are in the class of antibacterial peptides (67.9%), followed by new mechanism-associated peptides (17.3%), antiviral peptides (15.1%), antifungal peptides (14.0%), toxic peptides (7.9%), disease-associated peptides (6.7%), antiparasitic peptides (3.7%) and new functional peptides (0.5%) (Table 2).

#### Cross-linking update and new statistics

**Figure 2:**
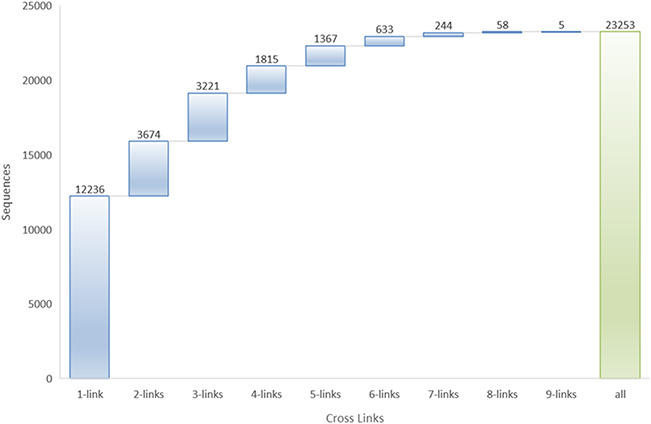
Cross-links to other databases in LAMP2.

Many peptides in LAMP2 belong to two or more databases. More than 45% of AMPs have two or more database links, and at most, one AMP sequence link to nine different databases (Figure 2).

**Figure 3:**
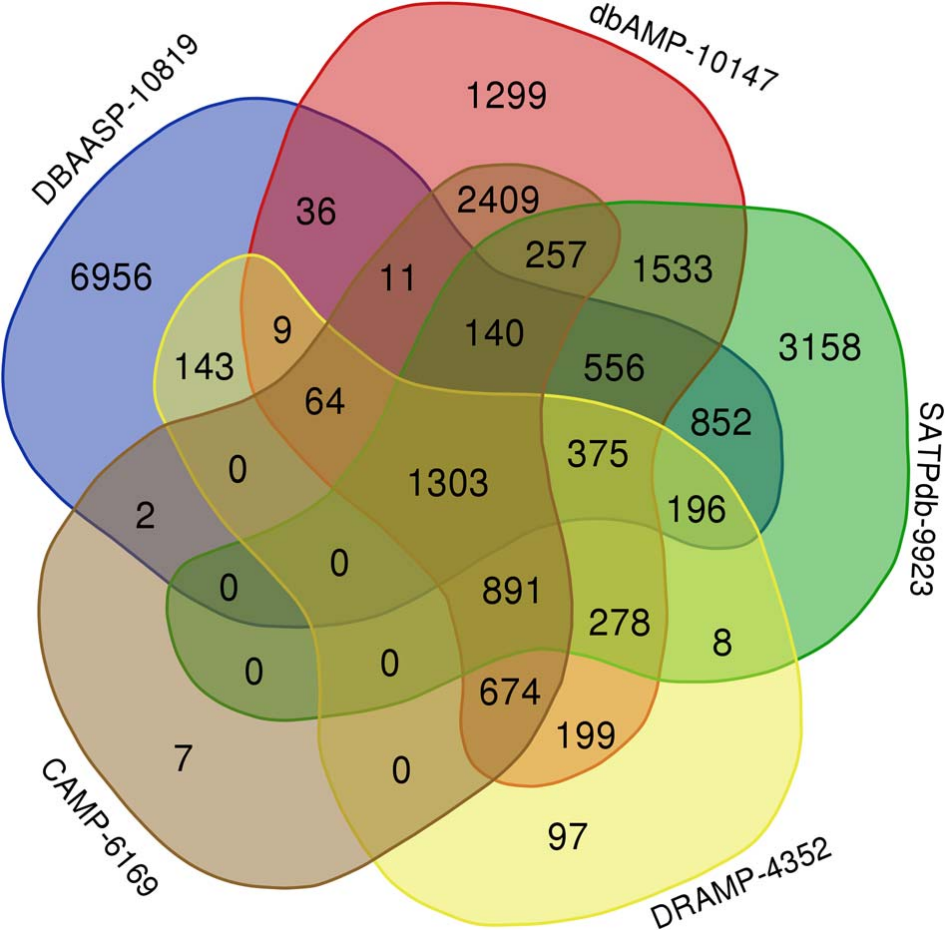
Venn diagram demonstrating peptides cross links among the top 5 databases in LAMP2 (plotted using http://bioinformatics.psb.ugent.be/webtools/Venn/).

**Figure 4:**
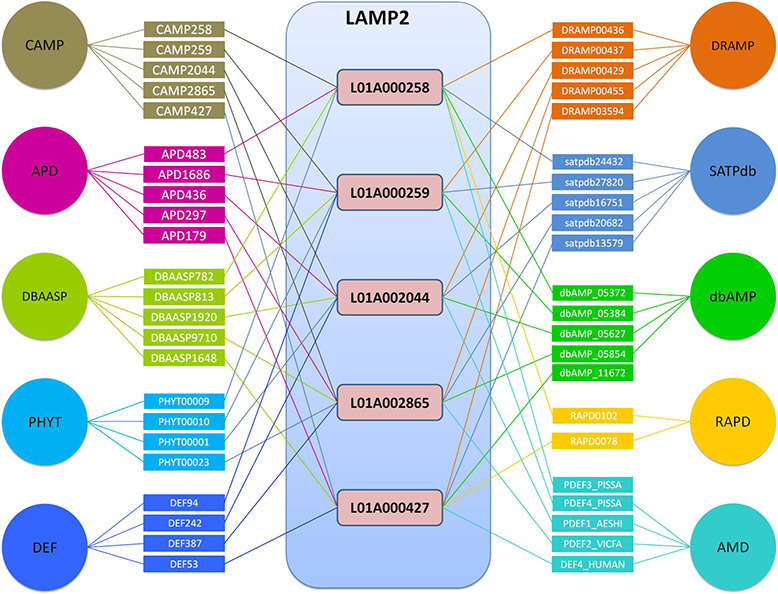
Top 5 AMPs in the number of cross-links in LAMP2.

The top 5 links to databases were 10 819 links to the DBAASP, 10 147 links to dbAMP, 9923 links to SATPdb, 6169 links to the CAMP and 4352 links to DRAMP (Figure 1). The cross-links among these top 5 databases have been demonstrated by Venn diagram (Figure 3). Although we have included almost all of the important databases so far, there are still more than 50% (12 236) sequences only link to a single database, indicating that these AMP sequences are somewhat unique or not representative and require further research (Figure 2). At the same time, it also shows that the databases holding these sequences are created on their own and are not universal. The top 5 AMPs in the number of cross-links are listed in Figure 4. More links often demonstrate the more comprehensive, complete information about the sequence.

### Utility of database

LAMP2 can be used to get exhaustive information of AMP on a single platform. For example, input ‘defensing’ in the protein name inputbox given at database search page. By a simple click on the search button, you will be directed toward a list of 30 entries stored in LAMP2, which are differentiated with a unique ID. A click on each ID will direct to a detailed display page, providing all the information about general information (LAMP_ID, name, full name, source, mass, sequence, sequence length, isoelectric point, activity and function), structure, activity (MICs), toxicity and reference. LAMP2 contains almost all the data currently included in the commonly used published databases. Take DEF1_PEA as an example: cross-linking provides hyperlinks to other public databases, such as SWISS P8192, CAMP 258, AMD PDEF3_PISSA, PHY PHYT00009, RAP RAPD0102, APD 00483, DBAASP782, dbAMP_05372, DRAMP00436 and SATPdb24432, which allows additional information on the AMP to be easily obtained. For example, if you are interested in family signatures of DEF1_PEA, you can click on the hyperlink CAMP 258. Or you want to know other physical-chemical parameters, you can click on the hyperlink DBAASP 782. Databases that did not appear above basically indicate that they did not include the AMP sequence. Topview function provides the top similar AMPs produced by the BLASTP program. Equipped with the detailed antimicrobial activity and cytotoxicity data, the cross-linking and Topview functions will serve the study of sequence-activity better.

More functions, statistical description and findings were described in our original article (15). Here, we take a successful example of utilizing the database in a real drug discovery. We previously investigated the LAMP database and selected CP-P (lamp-id: L08AP00001) as a template and designed a series of its derivatives considering structure-activity relationship (α-helical structure and hydrophobicity for peptide activity) and AMP statistics (the amino acid composition of AMPs, average net charge, sequence length) after using the Topview function in LAMP. Among them, the derivative peptide K11 exhibited low MICs (less than 10 μg/mL) and broad spectrum antimicrobial activity, especially exhibited strong therapeutic effect on antibiotic resistant clinical isolates of both Gram-positive and Gram-negative bacteria (25).

## Conclusion

LAMP2 currently holds the most entries (a total of 23 253 unique AMPs, until November 2019) and is the unique cross-linking AMP database. All entries in LAMP2 are cross-linked with individual databases to provide an option for easy switching to the individual databases for extensive insight to them. The updated database includes details about primary structure, collection, composition, source and function, especially highlighting 8 major functional classes and 38 functional activities. The updated version will be helpful to the scientific community.
